# Hsc70-4 aggravates PolyQ-mediated neurodegeneration by modulating NF-κB mediated immune response in *Drosophila*

**DOI:** 10.3389/fnmol.2022.857257

**Published:** 2022-11-08

**Authors:** Saurabh Rai, Madhu G. Tapadia

**Affiliations:** Cytogenetics Laboratory, Department of Zoology, Banaras Hindu University, Varanasi, Uttar Pradesh, India

**Keywords:** HSPA8, PolyQ, Neurodegeneration, NF-κB, Relish, Immune response, Chaperone, Hsc70-4

## Abstract

Huntington’s disease occurs when the stretch of CAG repeats in exon 1 of the *huntingtin* (*htt*) gene crosses the permissible limit, causing the mutated protein (mHtt) to form insoluble aggregates or inclusion bodies. These aggregates are non-typically associated with various essential proteins in the cells, thus disrupting cellular homeostasis. The cells try to bring back normalcy by synthesizing evolutionary conserved cellular chaperones, and Hsp70 is one of the families of heat shock proteins that has a significant part in this, which comprises of heat-inducible and cognate forms. Here, we demonstrate that the heat shock cognate (Hsc70) isoform, Hsc70-4/HSPA8, has a distinct role in polyglutamate (PolyQ)-mediated pathogenicity, and its expression is enhanced in the *polyQ* conditions in *Drosophila*. Downregulation of *hsc70-4* rescues PolyQ pathogenicity with a notable improvement in the ommatidia arrangement and near-normal restoration of optic neurons leading to improvement in phototaxis response. Reduced *hsc70-4* also attenuates the augmented immune response by decreasing the expression of NF-κB and the antimicrobial peptides, along with that JNK overactivation is also restored. These lead to the rescue of the photoreceptor cells, indicating a decrease in the caspase activity, thus reverting the PolyQ pathogenicity. At the molecular level, we show the interaction between Hsc70-4, Polyglutamine aggregates, and NF-κB, which may be responsible for the dysregulation of signaling molecules in *polyQ* conditions. Thus, the present data provides a functional link between Hsc70-4 and NF-κB under *polyQ* conditions.

## Introduction

Neurodegenerative disorders are incurable and characterized by the progressive loss of structure and function of the neurons. It leads to pathophysiological conditions, causing the loss of motor and cognitive activities, which worsens with age progression. Polyglutamine diseases, including Huntington’s, are caused due to the expansion of CAG repeats in genes resulting in the expansion of glutamate residues (Chung et al., 1993; Imbert et al., 1996; David et al., 1997; Gusella and MacDonald, 2000). The enormous increase in the size of the proteins lead to the formation of water-insoluble aggregates called inclusion bodies (IBs) (Skinner et al., 1997; Cummings et al., 1998; Warrick et al., 1998, 1999; Bence et al., 2001; Siska et al., 2015), which sequester the signaling molecules and alter the orchestrated pattern of various signaling processes impacting cellular homeostasis (Schaffar et al., 2004; Hodges et al., 2006; Benn et al., 2008). In Huntington’s disease, aggregates enter the nucleus and bind with the erroneous DNA sequences, some of which are response elements of p53 and thyroid hormone receptors, which enhances the pathogenesis (Steffan et al., 2001; Yohrling et al., 2003). IBs are also associated with vesicular proteins and cytoskeleton, and affect vesicular trafficking, axonal transport, and endocytosis (Poirier et al., 2002; Caviston et al., 2007).

Proteinopathies cause cellular stress which induces the production of evolutionarily conserved heat shock proteins (HSPs) like HSP70, HSP40, HSP90, and HSP60, which help them either by refolding them correctly or by tagging them for ubiquitin-mediated proteasomal degradation. The expanded PolyQ proteins bind with the ATPase domain of these chaperones and hamper their chaperonin activities. Initially, different HSPs are activated in response to the aggregates (Carmichael et al., 2000; Krobitsch and Lindquist, 2000; Söti and Csermely, 2002), but with an excess of aggregate protein load, ATPase activity of HSPs gets affected, thus impeding aggregate clearance (Soo et al., 2008). Heat-inducible *hsp70* and its cognate *hsc70* are often mentioned together due to their high sequence similarity. However, studies recently demonstrated that despite their sequence similarity, they associate with different client proteins in amyotrophic lateral sclerosis (ALS)-associated superoxide dismutase 1 (SOD1) disorder, indicating that apart from their preference for unfolded proteins, they show distinct outcomes (Ryu et al., 2020). Studies performed in the *in vivo* and *in vitro* models have mentioned that overexpression of *hsp70/hsc70* results in the reduction of *polyQ*-mediated aggregates (Novoselova et al., 2005; Evans et al., 2010; Thiruvalluvan et al., 2020). Contrary to this, the role of *hsc70* has also been shown in the progression of misfolded protein aggregation and propagation in a chaperone–client protein-dependent manner (Hatakeyama et al., 2004; Fontaine et al., 2016). Similarly, other reports have also shown the role of Hsc70 in promoting the aggregation of misfolded proteins, and downregulating *hsc70* reduced the accumulation (Imler et al., 2019; Johnson et al., 2020).

The immune response is usually protective, but it can sometimes cause more harm if triggered erroneously. It happens in neurodegenerative diseases, which hyperactivates the immune response, resulting in the inflammation of neurons, aggravation of neuronal degeneration and death (Marcora and Kennedy, 2010; Ellwardt and Zipp, 2014; McManus and Heneka, 2017; Gelders et al., 2018). Studies in *Drosophila* have shown that the overactivation of innate immune response causes neurodegeneration (Cao et al., 2013; Heneka et al., 2014; Dubey and Tapadia, 2018). *Drosophila*, and all insects, ward off pathogenic attacks by triggering the humoral and cellular components of their innate immune response. The humoral response engages in the production of antimicrobial peptides (AMPs) through the Toll and the immunodeficiency (IMD) pathways, which are activated by the orthologs of NF-κB transcription factors, Dorsal/DIF, and Relish, respectively. Regulation of inflammatory response upon neuronal injury is mediated partly by the *hsp70* gene family (Merkling et al., 2015). *Hsp70* (expressed from *HSPA1A/B* genes) and *hsc70* (expressed from *HSPA8* gene) shared almost 85% similarity and were presumed to have similar roles in the cells, but recent pieces of evidence suggest that they may have distinct functions (Ahn et al., 2005; Chuang et al., 2015). Deletion of *hsp70* genes makes mouse models sensitive to only stress but does not affect viability and fertility. On the other hand, deletion of *HSPA8* is lethal in vertebrates (Hunt et al., 2004; Kabani and Martineau, 2008), and human *HSPA8* but not *HSPA1A* can compensate for the loss of *SSA1-4 HSP70* genes in yeast cells (Tutar et al., 2006), suggesting distinct properties of these two chaperones.

With this information, we aimed to look at the specific role of heat shock cognate protein in *polyQ-*mediated pathogenesis in *Drosophila* and its effect on neurodegeneration and immune response. The *Drosophila* homolog of human *HSPA8* is *hsc70-4*. We show that among the different *hsc70* isoforms, *hsc70-4* is maximally induced, and its downregulation seems to impart significant morphological and functional rescue. We show that PolyQ aggregates physically interact with Hsc70-4 and Relish, leading to increased transcriptional activity and enhanced AMP expression. Enhanced JNK activity in *polyQ* condition gets consequently reduced following *hsc70-4* downregulation. Collectively, the present study demonstrates a functional role of Hsc70-4 for Relish activation in proteinopathies involving CAG repeats.

## Materials and methods

### Fly stocks and genetics

All *Drosophila* stocks were maintained on the standard food media at 25°C, and the genetic crosses were performed at standard lab conditions at a set temperature of 25°C. Fly stocks used in the present study are *Oregon-R^+^*, *UAS-Hsc70-4RNAi* (BL-28709 and BL-34836), *UAS-Hsc70-4WT* (BL-5846), obtained from Bloomington Drosophila Stock Center; *w^1118^; UAS-127Q.HA;* +/+, and *w^1118^; UAS-20Q.HA;* +/+ (Kazemi-Esfarjani and Benzer, 2000), *w^1118^;GMR-GAL4;* +/+ (Freeman, 1996), *w^1118^;GMR-GAL4:UAS-127Q/CyOGFP;* +/+ (Yadav and Tapadia, 2013), *w^1118^; UAS-httex1PQ93/CyO;* +/+ (Steffan et al., 2001).

### Photomicrographs and nail polish imprint of the adult eye

To examine the external morphology of adult eyes, flies of desired genotypes were etherized and their eyes were photographed using a Sony Digital Camera (DSC-75) attached to Nikon PDSL-42 stereo binocular microscope.

For nail polish imprint (Arya and Lakhotia, 2006), flies of the desired genotype were etherized followed by decapitation of their head with a needle. Decapitated heads were dipped in the transparent nail polish and then allowed to dry at room temperature. Then, the dry nail polish was peeled off with the help of a needle to create an exact replica of the external surface of the *Drosophila* eye, which was subsequently observed under the DIC optics in a Nikon Ellipse 800 microscope.

### Phototaxis assay

This assay was performed using Y-maze. The flies of the desired genotype were subjected to a phototaxis assay. A group of 10 flies were put in the Y-maze. One side of the arm was dark and was covered with black paper while another side of the arm was illuminated by external light, and the stem of the Y-maze was also covered with black paper (Singh et al., 2014). Flies were transferred into the stem of the Y-maze. After 60 s, the number of flies present in each arm was counted. These experiments were performed in triplicate for each genotype having 100 flies. Flies of light and dark arms were calculated into mean valves of percentage and presented as ± standard error mean.

### Immunostaining, antibodies, and confocal imaging

Eye imaginal discs of the desired genotypes were dissected out in 1X PBS followed by 20 min of 4% PFA fixation. Then, tissues were washed in 0.1% PBST three times (1× PBS, 0.1% Triton-X) for 10 min and then kept in blocking solution (1× PBS, 0.1% Triton-X, 0.1% BSA, 10% FCS, and 0.02% Thiomersal) for 2 h at RT followed by the overnight incubation of the primary antibody at 4°C. Post-incubation, the tissues were subjected to three times washing in 0.1% PBST for 10 min followed by blocking for 1 h and then incubated in a secondary antibody for 2 h at RT followed by washing three times in 0.1% PBST, counterstained with DAPI for 20 min, rinsed in 0.1% PBST, and finally mounted in glycerol containing an antifading agent, DABCO (Sigma, D8001).

Primary antibodies which were used in the experiments are rabbit anti-HA (1:1500, Sigma-H6908), rat anti-ELAV (1:100, DSHB-7E8A10), mouse anti-Futsch (1:100, DSHB-22C10), mouse anti-Dlg1 (1:20, DSHB-4F3), rabbit p-JNK (1:100, Promega-pTPpY), mouse anti-Relish (1:50, DSHB-21F3), rat anti-HSP70 (1:500, Sigma-SAB5200204), mouse anti-Hsc70 (1:100, Abnova-MAB6130), mouse anti-beta-tubulin (1:500, DSHB-E7), acridine orange (10 μg/ml, Sigma-A6014), and DAPI (1 mg/ml, Sigma-D9542). Imaging was done using Zeiss LSM 510 Meta confocal microscope. Images were processed with Adobe Photoshop7.

### Western blotting

Protein samples were prepared by dissecting out eye discs from wandering third instar larvae from the desired genotypes. Eye discs were dissected in 1× PBS, after which they were transferred in RIPA buffer (100 mM Tris-Cl pH 6.8, 100 mM NaCl, NP-40, EDTA, NaF, NaO_2_V_4_, 20% glycerol, protease inhibitor cocktail, and 2 mM PMSF). Then, the tissues were homogenized in ice and then subjected to centrifugation at 12,000 rpm for 20 min to collect the protein-containing supernatants. Then, protein quantification was done using Bradford’s protein quantification technique. Finally, 25 μg of protein were taken from the supernatant and added in a 1:1 ratio with sample buffer (100 mM Tris-Cl pH 6.8, 4% SDS, 0.2% bromophenol blue, 20% glycerol, 100 mM DTT, and 2 mM PMSF). Then, samples were denatured in a boiling water bath for 5 min and were centrifuged at 12,000 rpm for 10 min. After this, the supernatant was collected and subjected to SDS-PAGE. Protein samples were then transferred onto PVDF membrane using wet electrotransfer apparatus at 100 V for 1.5 h at 4°C. After the completion of the transfer, PVDF membranes were incubated in blocking solution (4% BSA in 0.1% TBST) for 2 h followed by primary antibody incubation for overnight at 4°C. Membranes were then washed in 0.1% TBST for 15 min in a repetition of three times. After this, membranes were incubated in HRP-tagged secondary antibody for 2 h at room temperature followed by three times washing with 0.1% TBST for 15 min each. Signals were detected by using ECL *via* ChemiDoc (VILBER).

### Immunoprecipitation

Eye imaginal discs were dissected in 1× PBS and then were collected in RIPA buffer (50 mM Tris-Cl pH 7.4, 150 mM NaCl, 1% NP-40, 0.1% SDS, 1 mM EDTA pH 8, 1 mM PMSF) accompanied with 1 μg/ml w/v protease inhibitor cocktail. Then, homogenized in ice and after centrifugation at 5,000 *g* at 4°C for 5 min, supernatants were quantified. Then, 1 mg of total proteins were taken and incubated with Protein G-Dynabeads (Invitrogen-10004D) at 4°C overnight. Then, beads were separated from the supernatant using a magnet stand and were subjected to washing in chilled RIPA buffer three times at 4°C for 5 min each. Then, beads were separated from the washing solution completely and subjected to elution. Protein elution was done in SDS sample buffer (100 mM Tris-Cl pH 6.8, 4% SDS, 0.2% bromophenol blue, 20% glycerol, 100 mM DTT, and 2 mM PMSF) and was denatured by boiling it for 5–10 min. Finally, beads were separated and the supernatant was subjected to Western blotting.

### Scanning electron microscopy

Head of 5-day-old flies were chopped and washed in 1× phosphate buffer (PB) and then fixed in Karnovsky’s fixative for 2 h at RT, after which head was post-fixed in 1% Osmium tetroxide in 0.1M PB, pH 7.4 for 1 h followed by three washes with 0.1 PB, pH 7.4, 15 min each at RT. The sample was then dehydrated in a graded series of ethanol (30, 50, 70, 90, 95, and 100%) and then air-dried. The dried samples were then mounted on the carbon-taped SEM stub and sputter-coated with the platinum. The SEM images were taken using a Carl Zeiss, EVO-18, scanning electron microscope.

### Quantitative RT-PCR

Total RNA was isolated from the eye imaginal discs of healthy wandering third instar larvae of the desired genotypes in order to check the expression pattern of several immune genes, such as *relish, attacin, cecropin, diptericin, drosocin, drosomycin*, and different isoforms of *hsc70* and *hsf.* In order to achieve so, wandering third instar larvae of the desired genotypes were dissected to isolate total RNA using TRIzol reagents by following the recommended protocol provided by Ambion, India. The RNA pellets were resuspended in 15 μl of DEPC-MQ, and after the pellets were dissolved, their quantitative estimation was done using spectrophotometric analysis. Then, 1 μg of each RNA sample was incubated with 1U of RNase-free DNaseI (Roche, Sigma-4716728001) for 30 min at 37°C to remove any residue DNA. Then, first-strand cDNA was synthesized from these incubated samples, by following the standard cDNA preparation protocol. The prepared cDNA was subjected to real-time PCR using forward and reverse primer pairs of target genes. Real-time PCR was done by using 5 μl of qPCR master mix (SYBER Green, Puregene, Genetix), 2 picoM/μl of each primer per reaction in 10 μl of the final volume in the ABI QuantStudio Real-Time PCR machine. To analyze the change in the expression, the relative fold change mRNA expression of different genes was calculated using ^ΔΔ^Ct value and then represented as ± standard error mean. Data normalization was done using *rp49* as an internal control. Sequences of primers used in this study are mentioned in Supplementary information 1.

### Statistical analyses

All statistical analyses were done using Prism software using either unpaired Student’s *t*-test to determine the significance between two independent variables or one-way ANOVA along with a suitable post-test in order to determine the significance in the variance of means between the different genotypes. Data are presented as ± standard error mean as values taken from three independent experiments. *P* values ≤ 0.05 (*) were considered statistically significant.

## Results

### *hsc70-*4 transcription gets maximally elevated in *polyQ*-overexpressed conditions and downregulation rescues the eye phenotype

**FIGURE 1:**
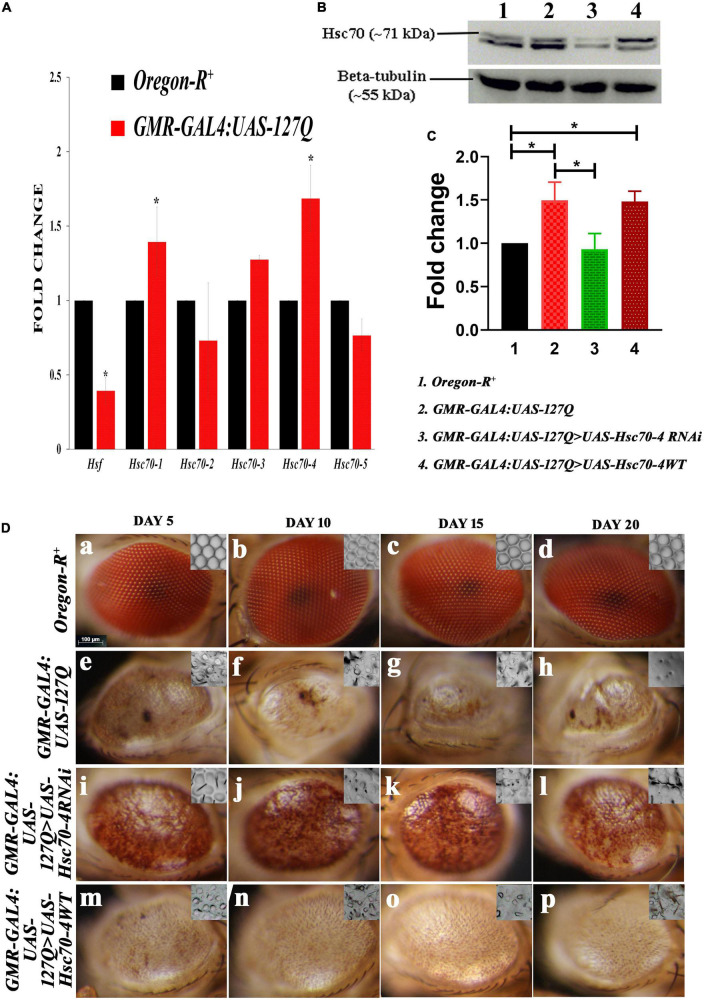
Alterations in the *hsc70* gene isoforms in the *polyQ*-overexpressing eye phenotype. **(A)** Transcript expressions of *hsc70-1, hsc70-3*, and *hsc70-4* are upregulated compared to *hsc70-2* and *hsc70-5*, while that of *hsf* is reduced in *GMR-GAL4:UAS-127Q* in comparison to *Oregon-R^+^*(normalization is done using *rp49*). **(B)** Western blot showing the expression of Hsc70 in different genotypes. **(C)** Graph shows quantitative analysis of Hsc70 expression normalized to beta-tubulin, showing significant upregulation of the protein in lane 2 and 4 compared to lane 1. In contrast, the protein level of Hsc70 in the lane 3 was comparable to lane 1. However, it was significantly downregulated compared to lane 2 (*n* = 3 and data shown as ±SEM and * indicated significance at the *p*-value of ≤0.05). **(D)** Eye degeneration in *GMR-GAL4:UAS-127Q* background is rescued after the downregulation of *hsc70-4*. Normal adult eyes in *Oregon-R^+^* **(a–d)**, disrupted in *GMR-GAL4:UAS-127Q* flies **(e–h)**, as they show distorted ommatidial pattern along with the loss of eye pigmentation due to expanded PolyQ aggregates accumulation. Downregulating *hsc70-4* restores the ommatidial arrangement and eye pigmentation in *polyQ-*driven flies **(i–l)**, while overexpressing *hsc70-4* does not correct the ommatidial arrangement and eye pigmentation in *polyQ*-driven flies **(m–p)**. The nail polish imprint of adult eyes showing the ommatidia pattern is in the inset of each image in this panel (scale bar 100 μm).

Huntington phenotype in *Drosophila* was obtained using the *UAS-GAL4* system (Brand and Perrimon, 1993) for spatiotemporal control of any transgene. Here, we used an eye-specific *GAL-4* driver, *GMR-GAL4*, to express 127 glutamate residues under the *UAS* promoter in the rhabdomeres, the neuronal cells of the *Drosophila* compound eye. Alteration in the transcription of various *hsp70* gene families has been reported in neurodegenerative diseases (Shieh and Bonini, 2011; Malik et al., 2013), but a lack of information about the expression of alternatively spliced transcript variants encoding different *hsc70* isoforms prompted us to check their expression. *Hsc70* has five isoforms, namely, *hsc70-1, hsc70-2, hsc70-3, hsc70-4* and *hsc70-5* in *Drosophila*. Expression of all five isoforms was assessed by RT-PCR using isoform-specific primers in three independent experiments, followed by Student’s *t*-test to check the significance level of the transcript’s fold change, following which *hsc70-1*, *hsc70-3*, and *hsc70-4* were found upregulated in *GMR-GAL4:UAS-127Q* background (Figure 1A), where *hsc70-4* gene expression was increased with the highest value of significance. No significant change was observed for *hsc70-2* and *hsc70-*5 isoforms in *polyQ-*expressing progenies.

Heat shock factor 1 (*Hsf1*) is activated by physiological stresses and activates heat shock genes required to mitigate stress. Studies have shown that activation of HSF1 in mammalian cells requires interaction with Hsc70 (Ahn et al., 2005), so we checked the expression of *hsf* in *Drosophila*, which was found to be downregulated. This observation suggests that under the *polyQ* condition, *hsf* is probably not involved in the regulation of *hsc70* expression. As the aggregate proteins impair the HSF-mediated heat shock response (HSR) induction in diseased conditions (San Gil et al., 2020), and a recent study has shown that *hsc70* is involved in the progression of the epithelial tumor in *a hsf-*independent manner (Singh et al., 2022). Therefore, these pieces of information suggest that the overexpression of these *hsc70* isoforms might be independent of *hsf* in the *polyQ*-expressed condition.

Since the transcriptional activity of *hsc70-4* was altered in the *polyQ* background, we looked at the protein levels of Hsc70. In the Western blot we detected two protein bands around 70kDa, the lower one was at ∼68 kDa, which is inducible Hsp70. It showed a typical pattern of elevated levels of Hsp70 in *polyQ* conditions in comparison to the wild-type. It also remained elevated in *hsc70-4* overexpressed *polyQ* background, but downregulating *hsc70-4* reduced its expression (in Supplementary information 4C). In the case of cognate Hsc70 (the upper band at ∼71 kDa), a significant upregulation of Hsc70 in the *polyQ* background was observed, which was reduced after expressing *hsc70-4 RNAi* in the *polyQ* condition. While the level of Hsc70 elevates after overexpressing *hsc70-4* in the *polyQ* condition, the upregulation was not significant in comparison to the *polyQ* condition alone (Figure 1B). This observation was validated after calculating the fold changes in the Hsc70 from three independent experiments, and statistical significance was determined by using one-way ANOVA followed by Tukey’s multiple comparisons test (Figure 1C).

To find if constitutively expressed heat shock protein augments the *polyQ* degenerative phenotype, we downregulated it using *UAS-Hsc70-4RNAi* in *GMR-GAL4:UAS-127Q* background, and the eye phenotype of adult flies was observed. The compound eyes of *Drosophila* have approximately 800 repeating unit eyes or ommatidia that are organized into a hexagonal array and contain an invariant number of photoreceptor neurons and accessory cells that can be unambiguously identified by their position within the ommatidium. The typical array of hexagonal lattice in the adult eyes exhibited loss of ommatidial clusters and regular spacing because of multiple fusions after *polyQ* misexpression (Figures 1D, e–h), as compared to the wild-type eyes (Figures 1D, a–d). A significant improvement in the eye pigmentation and ommatidial arrangement of *GMR-GAL4:UAS-127Q* > *UAS-Hsc70-4RNAi* adult flies was observed, with a remarkable restoration of pigmentation loss and ommatidial disruption, even at the age of 20 days (Figures 1D, i–l), in comparison to *GMR-GAL4:UAS-127Q* flies (Figures 1D, e–h), where, along with the pigmentation loss and ommatidial disruption, there was a gradual reduction in eye size from 5-day-old to 20-day-old flies. In *GMR-GAL4:UAS-127Q* > *UAS-Hsc70-4WT*, no restoration in the eye pigmentation or ommatidial pattern of the degenerated eyes was observed (Figures 1D, m–p), further suggesting that only downregulation of *hsc70-4* will significantly rescue the degenerated eye phenotype in *polyQ* background.

**FIGURE 2:**
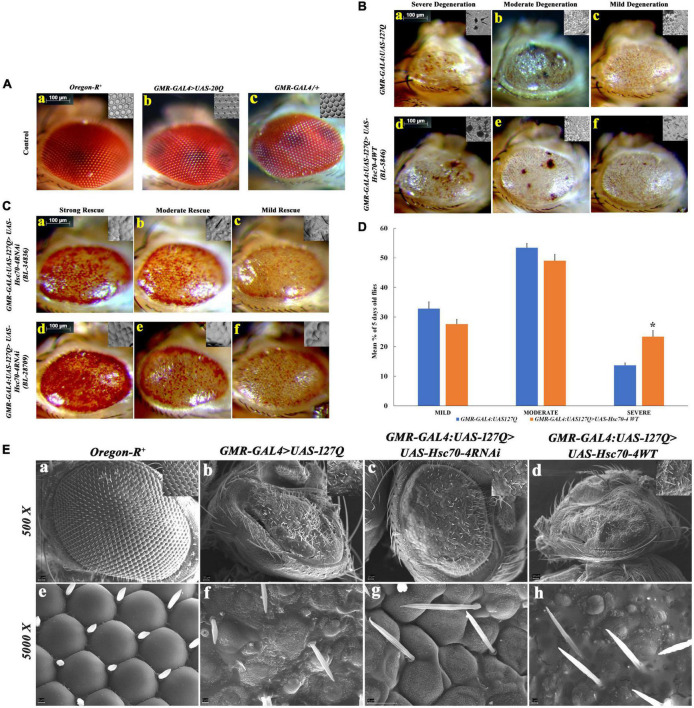
Detailed morphological observations of eye phenotype in *hsc70-4* regulated condition in 5-day-old *polyQ-*expressing flies. **(A)** Eye phenotype and ommatidial arrangement of three control genotypes: **(a)** *Oregon-R^+^*; **(b)** *GMR-GAL4:UAS-20Q*; and **(c)**, *GMR-GAL4*/+. *Oregon-R^+^* is the negative control, *GMR-GAL4*/+ is a positive control, and *GMR*-*GAL4:UAS-20Q* is also an experimental control. Both of their eye phenotypes are very similar to that of the negative control group. **(B)** Degeneration of the eye phenotype is categorized into three groups, and based on this categorization, the frequency of eye degeneration is compared between *polyQ*-expressing flies and *hsc70-4* overexpressing *polyQ* flies. These are (i) mild degeneration in panels **(c,f)** shows less roughening in the eye (with lesions only at the periphery); (ii) moderate level of degeneration in panels **(b,e)** where degeneration and necrotic patches are present in either of the eyes; and (iii) severe degeneration in panels **(a,d)** where the eye size is smaller than the mild eye phenotype along with larger necrotic patches (scale bar 100 μm). **(C)** Rescue in the eye phenotype after the downregulation of *hsc70-4* in *GMR-GAL4:UAS-127Q* is checked using two different RNAi lines. The rescue phenotype is categorized into three groups: (i) mild rescue phenotype in panels **(c,f)** shows recovery in the eye pigmentation at the margin and slight improvement in the ommatidial arrangements. (ii) Moderate rescue in panels **(b,e)** leads to the recovery of the loss of pigmentation throughout the eye and the recovered ommatidia. (iii) High rescue phenotype in panels **(a,d)** where eye pigmentation is very prominent along with a highly recovered ommatidial pattern (scale bar 100 μm). **(D)** The graph shows the severity of eye degeneration change upon overexpressing the *hsc70-4* in the *GMR-GAL4:UAS-127Q* background. Slight changes in the percentage of flies with either mild and moderate degenerating eye phenotype have been observed between *GMR-GAL4:UAS-127Q* and *GMR-GAL4:UAS-127Q*> *UAS-Hsc70-4WT*, whereas a significant increase in the percentage of the severe eye phenotype bearing 5-day-old flies which suggests an increase in ocular degeneration after overexpressing *hsc70-4*. The eye phenotype of each genotype was observed after three independent crosses and n ≥ 100, and data are shown as ±SEM (* indicated significance at the *p*-value of ≤0.05). **(E)** SEM image panels show the eye phenotype of different genotypes in detail. **(a,e)** *Oregon-R^+^*, **(b,f)** *GMR-GAL4:UAS-127Q*, **(c,g)** *GMR-GAL4:UAS-127Q*> *UAS-Hsc70-4RNAi*, and **(d,h)** *GMR-GAL4:UAS-127Q*> *UAS-Hsc70-4WT* (scale bar 20 μm).

Before validating the results obtained with *hsc70-4 RNAi* (Figure 1D), we compared different internal controls to ensure that the results are specific for 127Q residues. For this, we compared the eye phenotype of wild-type with no PolyQ aggregates (Figures 2A, a), *GMR-GAL4>UAS:20Q.HA* with only 20 Polyglutamine residues (Figures 2A, b), and a single copy of *GMR-GAL4/+* (Figures 2A, c), and did not observe any significant difference among them, suggesting that the degenerated eye phenotype in *GMR-GAL4:UAS-127Q.HA* is only due to the expanded repeats of 127 glutamine. Next, we used two different RNAi lines of *hsc70-4* from BDSC (Bl-28709 and BL-34836) to downregulate *hsc70-4* and one overexpression line (BL-5846) in order to upregulate *hsc70-4* in *polyQ* background and compare the eye phenotype in the 5-day-old flies. The degenerating eye phenotype was categorized into three groups based on the severity of the morphological disruption in the eye, which were mild (Figures 2B, c,f), moderate (Figures 2B, a,e), and severe (Figures 2B, a,d) (Sanokawa-Akakura et al., 2010). After overexpressing *hsc70-4* in *GMR-GAL4:UAS-*127Q condition, the mean percentage of flies with severe eye phenotype increased significantly. However, the percentage of flies with either mild or moderate eye phenotypes remained unchanged (Figure 2D). These observations were made by observing the eye phenotype of 5-day-old flies from their respective genotype, and the mean percentage of those adult flies was recorded from three independent experiments (where n ≥ 100) followed by Student’s t-test in between each phenotypic category, to check for their significance level.

Likewise, two *hsc70-4* RNAi lines were used separately to downregulate it in *GMR-GAL4:UAS-*127Q background. Afterward, the transcript expression of *hsc70-*4 was checked in the same and compared to the *polyQ*, which was found to be significantly depleted, thus, validating the knockdown effect of these two RNAi lines (in Supplementary information 3). The resulting progenies showed the rescue in the degenerated eye phenotype, which was categorized further into three different groups—mild, moderate, and high—based on the strength of their phenotypic rescue. The mild rescued phenotype (Figures 2C, c,f) showed a slight improvement in the ommatidial arrangement and mild restoration of the pigmentation loss. Moderate rescued phenotype (Figures 2C, b, e) was demarcated as the recovery of eye pigmentation throughout the eye along with the recovered ommatidia arrangement, and strong rescued phenotype (Figures 2C, a,d) showed a remarkable improvement in the ommatidial arrangement and the recovery of the eye pigmentation.

After the phenotypic confirmation, we wanted to observe the eye surface topography in detail for the confirmation of the previously speculated rescued phenotype. So, we did the SEM imaging of the eye of the same genotypes. We observed severely disrupted eye morphology in the *polyQ* background, showing small asymmetrical ommatidia along with very few irregularly arranged mechanosensory bristles present (Figures 2E, b, f), in comparison to the wild-type eye (Figures 2E, a, e). The ommatidial disruption elevated in *polyQ* flies upon overexpressing *hsc70-4* (Figures 2E, d, h), as the ommatidia were scarcely present and were smaller in size, with even lesser mechanosensory bristles. However, after downregulating *hsc70-4* in *GMR-GAL4:UAS-127Q* background, the shape and arrangement of ommatidia were restored, as there was a clear boundary between the ommatidium and an increase in the abundance of mechanosensory bristles, which were in much more orderly fashion (Figures 2E, c, g). Thus, all these observations suggest that PolyQ aggregates impact neurodegeneration, which is probably associated with the dysregulation of *hsc70-4*, as downregulating it rescues the *polyQ-*mediated degenerated eye phenotype.

### Downregulation of *hsc70-4* improves the structural and functional phenotype of the *GMR-GAL4:UAS-127Q* progenies

The cell-type differentiation of eye cells begins midway through the third instars stages, with the appearance of the ‘morphogenetic furrow,’ and posterior to it, progressive induction of cell fate occurs, with the development of photoreceptor neurons/rhabdomeres (first neuron R8, then R2 and R5, R3/R4, R1/R6, and finally R7 within each ommatidium). Aggregate accumulation in *polyQ* background led to the disruption in these photoreceptor neurons, leading to the structural and functional loss in the aggregates-infested eye of the *Drosophila*.

**FIGURE 3:**
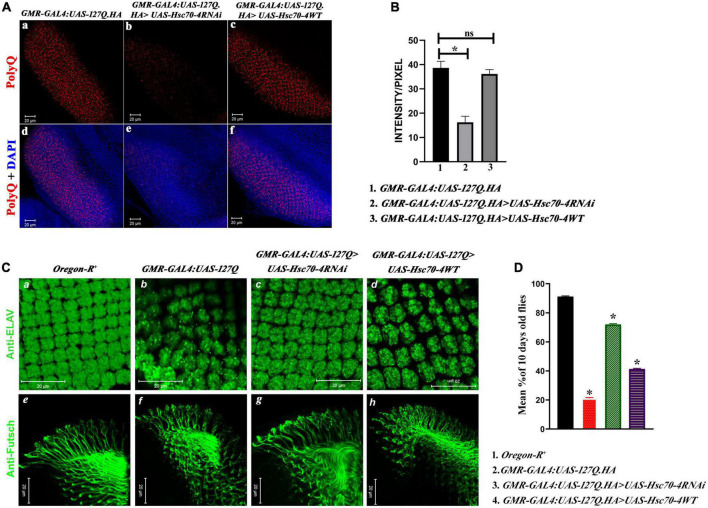
*hsc70-4* downregulation reduces the aggregates accumulation, resulting in the improved arrangement of rhabdomeres and optic neurons. Thus, it enhances the phototaxis response. **(A)** The panel shows the PolyQ aggregates (in red) accumulation in **(a)** *GMR-GAL4:UAS-127Q*; **(b)** *GMR-GAL4:UAS-127Q*>*UAS-Hsc70-4RNAi;* **(c)** *GMR-GAL4:UAS-127Q*> *UAS-Hsc70-4WT*. Panels **(d–f)** from the panel **(A)** show the nuclei staining (in blue) along with the aggregates of the respective genotypes (scale bar 20 μm). **(B)** Quantitative analysis of the intensity/pixel of the immunofluorescence images shows PolyQ aggregates accumulation in the different genotypes from three independent experiments and data shown as ±SEM (ns indicates insignificant change at *p*-value ≤ 0.05%, and * indicates the significance at a p-value of ≤ 0.05). **(C)** *hsc70-4* downregulation restores the rhabdomere arrangement and the axonal connection disruption in *GMR-GAL4:UAS-127Q*-driven flies. **(a,e)** *Oregon-R^+^*, **(b,f)** *GMR-GAL4:UAS-127Q*, **(c,g)** *GMR-GAL4:UAS-127Q*> *UAS-Hsc70-4RNAi*, **(d,h)** *GMR-GAL4:UAS-127Q*> *UAS-Hsc70-4WT*. Anti-ELAV (in green) to mark rhabdomere and 22C10 staining against Futsch (in green) to visualize the optic neurons (scale bar 20 μm). **(D)** The graph shows the phototaxis response in the 10-day-old flies of *Oregon-R^+^*, *GMR-GAL4:UAS-127Q*, *GMR-GAL4:UAS-127Q*> *UAS-Hsc70-4RNAi*, and *GMR-GAL4:UAS-127Q*> *UAS-Hsc70-4WT* (the experiment was performed in triplicate where *n* ≥100 for each genotype, and data are shown as ±SEM; * indicated significance at the *p*-value of ≤0.05%).

Having observed the phenotypic rescue after downregulating *hsc70-4*, we looked for the aggregates deposition in the eye imaginal discs of developing late third instar larvae and observed that the number and size of PolyQ aggregate decreased drastically in *hsc70-4* RNAi-driven *GMR-GAL4:UAS-127Q* progenies (Figures 3A, b, e) in comparison to undriven *GMR-GAL4:UAS-127Q* (Figures 3A, a,d). In contrast, no change in the aggregates level was observed in the case of *GMR-GAL4:UAS-127Q* > *UAS-Hsc70-4WT* genotype (Figures 3A, c, f) [this observation was supported by the quantitative analysis (Figure 3B) using the mean value of intensity/pixel of PolyQ aggregates measured from the immunofluorescence images of the respective genotype which was mentioned in Figure 3A]. Hence, the downregulation of *hsc70-4* in *GMR-GAL4:UAS-127Q* background led to a significant reduction in aggregates deposition in the developing eye disc.

To trace the effect of reduction in the PolyQ aggregates on degenerative eye phenotype, we checked the pattern of rhabdomeres, which can be easily visualized by ELAV staining, a pan-neuronal marker. Disruption in the rhabdomere pattern occurred due to polyglutamine toxicity in *GMR-GAL4:UAS-127Q*-driven conditions (Figures 3C, b). After downregulating *hsc70-4* in *polyQ*-overexpressing eye discs, the rhabdomere arrangement was restored (Figures 2C, c) and resembled wild-type (Figures 3C, a). The pattern of rhabdomeres after overexpressing *hsc70-4* remained disrupted (Figures 3C, d). We also checked the ommatidial arrangement using Dlg, a membrane marker, to validate previous observation (in Supplementary information 5A), which further validates the severe rhabdomere disruption after overexpressing *hsc70-4*. As the number of rhabdomeres reduces from seven in the wild-types to an average of four per ommatidium in *polyQ* condition, which furthers reduces to 3-rhabdomere/ommatidium in *GMR-GAL4:UAS-127Q* > *UAS-Hsc70-4WT* and downregulating, the *hsc70-4* recovers this loss to an average of > 5-rhabdomeres per ommatidial unit in the eye disc (in Supplementary information 5B). Therefore, downregulating *hsc70-4* restores the loss of rhabdomeres, thus leading to the recovery of the ommatidial disruption caused by *polyQ* background.

Axons of photoreceptor cells innervate into the optic lobe of the brain, and the proper connection of these axons is a hallmark of neuronal integrity (Andrews et al., 2010). Futsch is one of the microtubular-associated proteins present in the axons and can be used as an axonal marker to visualize the axonal connection. In the *polyQ* expressing eye disc of the third instar larvae, the axonal connections were disrupted severely, confirming that the entire morphology gets disrupted (Figures 3C, f) compared to the wild-type (Figures 3C, e). The axonal connections get restored in the eye disc of *GMR-GAL4:UAS-127Q* > *UAS-Hsc70-4RNAi* genotype (Figures 3C, g), but it remained disrupted in the case of *GMR-GAL4:UAS-127Q* > *UAS-Hsc70-4WT* genetic background (Figures 3C, h).

After visualizing the structural phenotypes, we checked the phototaxis response to investigate its functional implications. The phototaxis response was severely hampered in the *polyQ-*expressing eyes of 10-day-old flies. Less than 20% of the total adult population responded to the light, which was slightly improved after the overexpression of *hsc70-4.* However, the total percentage of flies responding to light remains less than half its population. On the other hand, downregulating *hsc70-4* restored the phototaxis response in more than 60% of the adult population (Figure 3D). Therefore, as the rhabdomere pattern and the neuronal cytoskeletal arrangement were rescued after downregulation of *hsc70-4*, it led to the restoration of normal cellular processes that includes maintenance of cell shape and morphology, cytokinesis, adhesion, migration, phagocytosis, and endocytosis (Pelissier et al., 2003; Riggs et al., 2003; Myers and Casanova, 2008). Thus, the structural rescue in *polyQ-*expressing condition that occurs after reducing the transcript expression of *hsc70-4* also leads to functional rescue.

### *hsc70-4* downregulation causes the reduction of the elevated immune response

**FIGURE 4:**
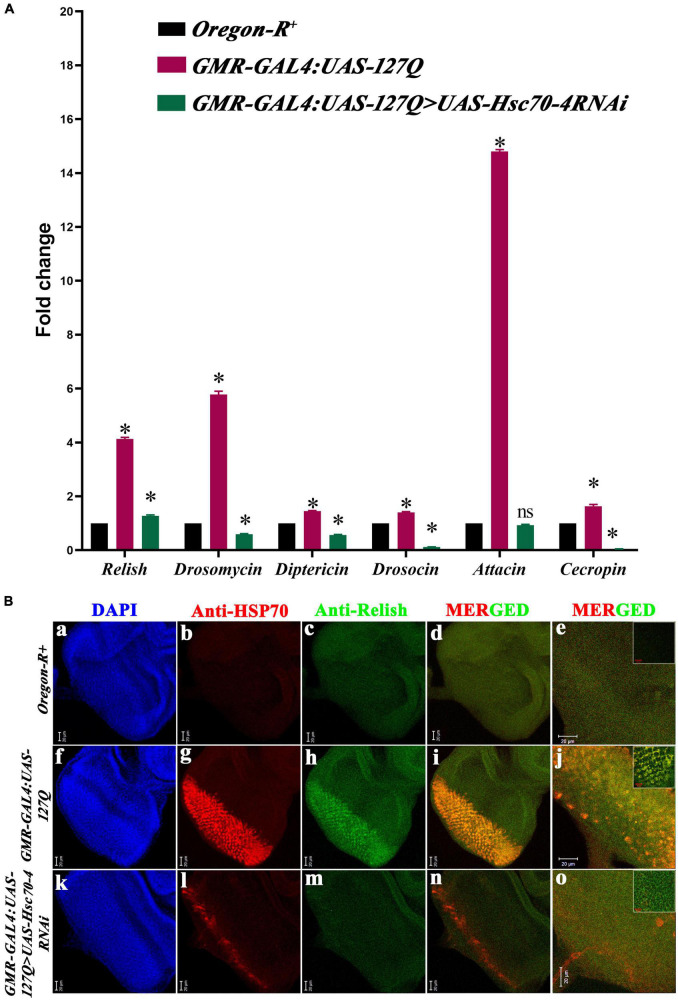
Elevated immune responses in the *polyQ* background get reduced upon *hsc70-4* downregulation. **(A)** The histogram shows qPCR analysis representing the fold change in transcript level of *relish* and *AMPs* in *GMR-GAL4:UAS-127Q* conditions significantly elevated in comparison to the wild-type. But *GMR-GAL4:UAS-127Q*> *UAS-Hsc70-4RNAi* shows a significant decline in these respective immune genes (data normalized to *rp49*, three biological replicated were used, and data are shown as ±SEM; ns means insignificant change at *p*-value of ≤0.05, and *indicated significance at the *p*-value of ≤0.05). **(B)** Reduction in expression of Relish (green) and HSP70 (red) after downregulation of *hsc70-4* in *GMR-GAL4:UAS-127Q>UAS-Hsc70-4RNAi* in comparison to *GMR-GAL4:UAS-127Q*, in lower magnification **(a–d,f–i,k–n)** and **(e,j,o)** shows the higher magnification (scale bar 20 μm). The inset in **(e,j,o)** was captured at a further 3X zoom to show co-localization (scale bar 10 μm). Genotype description, *Oregon-R^+^* **(a–e)**; *GMR-GAL4:UAS-127Q* **(f–j)**; *GMR-GAL4:UAS-127Q> UAS-Hsc70-4RNAi* **(k–o)**.

Previous studies have reported the elevation of immune response in neurodegenerative conditions, which aggravates the physiological complications of the disease (Cao et al., 2013; Heneka et al., 2014), and it has been shown in *Drosophila* that *polyQ* enhances immune response (Dubey and Tapadia, 2018). This dysregulation happens due to the accumulation of Relish, trapped by PolyQ aggregates (Takano and Gusella, 2002), which ultimately causes the hyperactivation of the immune response and elevates the expression of AMPs. Studies in human and mouse models have shown the involvement of HSPA8 in the activation of NF-κB, thus, regulating the level of the innate immune response (Sheppard et al., 2014; Kumada et al., 2019). As *hsc70-4* is the *Drosophila* ortholog of human *HSPA8*, we checked whether *hsc70-4* regulates the hyperactivated immune response in *polyQ-*mediated neurodegeneration. The transcript levels of *relish* and *AMPs* in *Oregon-R^+^* and *GMR-GAL4:UAS-127Q* were checked by qPCR and compared with the *GMR-GAL4:UAS-127Q* > *UAS-Hsc70-4RNAi* genotype. In comparison to *Oregon-R^+^*, the expression of *relish* and the different *AMPs* were significantly upregulated in *GMR-GAL4:UAS-127Q.* Maximum upregulation was observed in *attacin* and *drosomycin*, but *diptericin*, *drosocin*, and *cecropin* were upregulated too. Downregulating *hsc70-4* in *polyQ* background resulted in a significant reduction in gene expression of *relish* and all the *AMPs* (Figure 4A).

The C-terminal of Relish protein harbors 49kDa inhibitory ankyrin repeats, which are cleaved off to release the transcriptionally active nuclear localizing Rel homology domain. So, when we compared the expression of Relish in *GMR-GAL4:UAS-127Q* progenies (Figures 4B, h) to the *Oregon-R^+^* (Figures 4B, c), we found that it had significantly increased. After downregulating *hsc70-4* in *polyQ* background, the expression of Relish reduced (Figures 4B, m), suggesting a significant drop in the Relish activation and accumulation. We further looked at the expression of HSP70 to see if the enhanced inflammatory response in the *polyQ* background upsurges the stress-inducible HSP70, after which a significant surge was observed in the level of HSP70 in the *GMR-GAL4:UAS-127Q* background (Figures 4B, g) and was reduced upon downregulating *hsc70-4* in the same genetic background (Figures 4B, l). The quantitative analysis from the Western blot also validates the similar expression pattern of Hsp70 (in Supplementary information 4C). Furthermore, colocalization between these two proteins was detected after co-staining Relish and HSP70 in *polyQ* conditions (Figures 4B, i,j), which has also been reported earlier (Ran et al., 2004; Somensi et al., 2017). However, reduction in the expression of HSP70 and Relish upon downregulating *hsc70-4* also depleted the colocalization signal (Figures 4B, n,o), which might be happening because the elevation of Relish in *polyQ* condition caused the accumulation of HSP70 and reduction in the inflammation depletes the HSP70 accumulation. Thus, these results suggest that *hsc70-4* downregulation in the *polyQ*-expressing background may reduce hyperactivated innate immune response along with the reduction in the level of cellular stress.

### Polyglutamine aggregates physically interact with Hsc70 and regulate the activation of Relish

In continuation to the earlier observation, the question arises as to how alteration in the expression of *hsc70-4* in *polyQ*-overexpressed background affects the activation of Relish. Therefore, to find the answer, we looked into the possibility of the physical interaction of Hsc70 with Relish in *polyQ*-expressed conditions. Studies mention the role of Hsc70 in the clearance of aggregated proteins (Wyttenbach, 2004; Shieh and Bonini, 2011), physical interaction of Hsc70 with the polyglutamine stretch (Monsellier et al., 2015; Kim et al., 2016; Imler et al., 2019; Johnson et al., 2020), and polyglutamine aggregate interaction with NF-κB (Relish/Dorsal-Dif family) *via* HEAT-like motif and co-translocated into the nucleus (Takano and Gusella, 2002, Khoshnan et al., 2004). These studies suggest the possible role of polyglutamine aggregates in dysregulating both Hsc70 and NF-κB by physically interacting with both of them.

**FIGURE 5:**
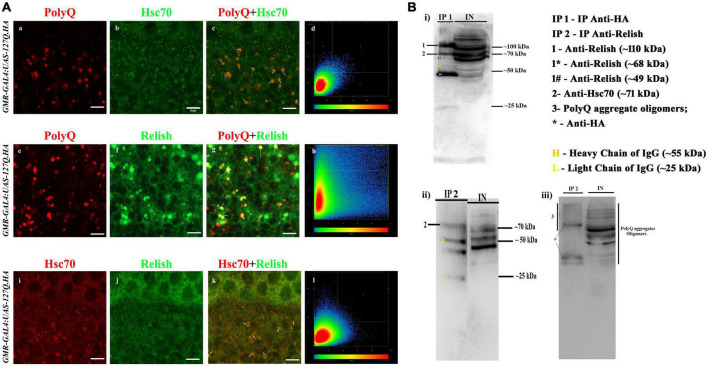
PolyQ aggregates colocalized and coprecipitated with Hsc70 and Relish. **(A)** The panel shows the section image of the eye imaginal disc of *GMR-GAL4 UAS 127Q* third instar larvae. **(a–d)** Co-staining of the PolyQ aggregates (in red) and Hsc70 (in green) shows that PolyQ aggregates and Hsc70 colocalize with each other; the arrow demarcates the colocalized regions in yellow (scale bar 5 μm). **(e–h)** Co-staining of the PolyQ aggregates (in red) and Relish (in green) shows that PolyQ aggregates colocalize with Relish. Arrow demarcates the colocalized regions in yellow (scale bar 5 μm). **(i–l)** Co-staining of Hsc70 (in red) and Relish (in green) co-stained in the *polyQ-*expressing eye disc shows the colocalization (in yellow), which is marked by the arrow (scale bar 5 μm). **(B)** Immunoblot panels illustrated the presence of the interacting proteins in the co-IP elute (denoted as IP) from the crude protein sample (demarcated as IN, means input) of *GMR-GAL4:UAS-127Q*.*HA* background. **(i)** Western blot shows the presence of Relish and Hsc70 in the IP1 sample after precipitation of PolyQ aggregate using anti-HA and the expression pattern in input. Images ii and iii are immunoblot showing the presence of Hsc70 and PolyQ aggregates, respectively, in the IP2 sample after precipitation of Relish using anti-Relish, and the expression pattern was compared with the input sample. IP 1—eluted protein sample after pull-down with anti-HA; IP 2—eluted protein sample after pull-down with anti-Relish; IN—crude protein sample of *GMR-GAL4:UAS-127Q.HA* genetic background; 1- whole-Relish protein (∼110 kDa), 1* donates a faint band of N-terminal Relish (∼68 kDa), and 1# denotes a sharp band of C-terminal Relish (∼49 kDa); 2—Hsc70 (∼71 kDa); 3—PolyQ aggregates oligomers detected by anti-HA along with *representing the bands of anti-HA; H and L denote heavy chain (∼55 kDa) and light chain (∼25 kDa) of IgG.

Therefore, we hypothesized that PolyQ-mediated dysregulation of NF-κB might be caused because of the hyperactivation of NF-κB by Hsc70. To confirm this, we needed to find out whether these three proteins interacted with each other in *polyQ*-overexpressed conditions. Thus, we performed a colocalization experiment in *GMR-GAL4:UAS-127Q.HA* background for PolyQ, Hsc70, and Relish, after which results showed colocalization between PolyQ and Hsc70 (Figures 5A, a–d), PolyQ and Relish (Figures 5A, e–h), and Relish and Hsc70 (Figures 5A, i–l) (yellow color in panel c indicates colocalization). Quantitative analysis of colocalization was done using Zen software along with the colocalization scattered plot (Figures 5A, d,h,l). Moreover, the overlap coefficient was also checked, which was ≥ 0.5, with a positive correlation of ≥ 0.1 in all three conditions (Figures 5A, c,g,k). Additionally, upon analyzing the surface-rendered image of the 3D projection of the eye disc stained for PolyQ, Hsc70, and Relish, the coappearance of Hsc70 and Relish was found within the PolyQ aggregate (data in the Supplementary information 6). Thus, these findings project the possibility of physical interaction of Relish and Hsc70 while PolyQ aggregates sequester both proteins.

For further confirmation of these interactions, we performed the co-immunoprecipitation experiment. To achieve this, we used anti-HA and anti-Relish antibodies to pull down PolyQ aggregates and Relish, respectively, from the protein sample extracted from the eye disc of third instars of *GMR-GAL4:UAS-127Q.HA* genetic background. Then, the interacting partners were detected by Western blotting of the eluted sample, denoted as the IP sample, and compared with the crude protein sample from *GMR-GAL4:UAS-127Q.HA* genetic background, which is represented as INPUT. After the immunoprecipitation of PolyQ aggregates using an anti-HA antibody, a band of ∼70 kDa in the blot of IP1 detected the Hsc70 labeled as **2** (Figures 5B, i). Also, three different isoforms of Relish were observed in the Western blot of the IP1, two sharp bands of whole-Relish protein labeled as **1** (∼110 kDa), C-Relish labeled as **1#** (∼49 kDa), and a very faint band of N-Relish labeled as 1* (∼68 kDa). Thus, it was confirmed that Hsc70 and Relish were coprecipitated with PolyQ aggregates in the IP1. Similarly, after the immunoprecipitation of Relish in *polyQ* background, a band of Hsc70 around ∼70 kDa in the blot of IP2 was detected (Figure 5B, ii), which is inferred as the physical interaction of Hsc70 with Relish. PolyQ aggregate oligomers were detected in the Western blot of the IP2 sample (Figures 5B, iii), where several bands with a smeary appearance were observed due to the presence of fragmented PolyQ oligomer during the processing along with the three clear bands labeled as * which might be the PolyQ trapped with the IgG present in elute sample of IP2 (Figures 5B, iii). Therefore, the co-immunoprecipitation experiments confirmed the possibility of co-interaction of PolyQ aggregates, Hsc70, and Relish in *polyQ*-expressing background, which highlights one of the underlying causes of Hsc70 dysregulation in *GMR-GAL4:UAS-127Q.HA* expressing background.

### Downregulation of *hsc70-4* in *GMR-GAL4:UAS-127Q* genetic background reduces the elevated p-JNK level

**FIGURE 6:**
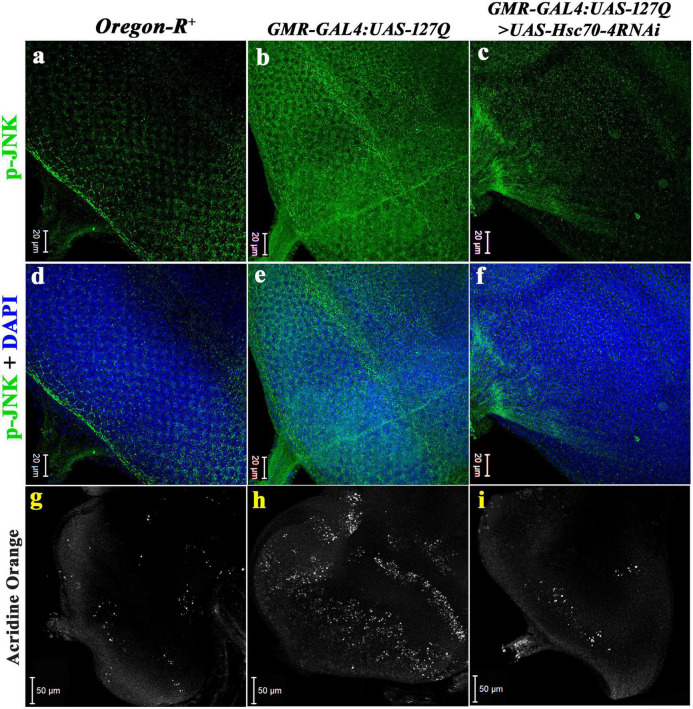
Downregulation of *hsc70-4* reduces the elevated p-JNK level in *polyQ-*overexpressed background, thus rescuing the cell death in the diseased eye disc. **(a)** The eye disc of *Oregon-R^+^* shows the typical expression pattern of p-JNK (in green), which drastically elevates in panel **(b)** *GMR-GAL4:UAS-127Q*, and **(c)** downregulating *hsc70-4* in the *polyQ-*overexpressed condition reduces the hyperactivated p-JNK. Panels **(d–f)** show p-JNK staining merged with DAPI in the eye disc of *Oregon-R^+^*, *GMR-GAL4:UAS-127Q*, and *GMR-GAL4:UAS-127Q*> *UAS-Hsc70-4RNAi*, respectively (scale bar 20 μm). White puncta in the images **(g–i)** are acridine orange stained dead cells in the eye disc of third instars of *Oregon-R^+^*, *GMR-GAL4:UAS-127Q*, and *GMR-GAL4:UAS-127Q*> *UAS-Hsc70-4RNAi*, respectively, (scale bar 50 μm).

c-Jun N-terminal kinase (JNK) is one of the central signaling cascades of the MAPK signaling pathway and functions as a regulator of cellular processes like proliferation, development, and apoptosis (Liu and Lin, 2005). So, we checked its expression in the *polyQ-*overexpressing eye discs. The level of activated JNK, i.e., p-JNK in the *polyQ-*overexpressing eye disc was considerably elevated in *GMR-GAL4:UAS-127Q* (Figures 6b,e), in comparison to *Oregon-R^+^* (Figures 6a,d), which is similar to an earlier report (Dubey and Tapadia, 2018), which reduced significantly in *GMR-GAL4:UAS-127Q* > *UAS-Hsc70-4RNAi* (Figures 6c,f). This suggests that the elevated p-JNK level in the *polyQ*-expressing condition restores normal upon *hsc70-4* downregulation. Quantitative analysis of the immunofluorescence images (data in the Supplementary information 7) further confirms the rescue in the p-JNK level after *hsc70-4* downregulation in the *polyQ* condition. More than a 15-fold reduction in the p-JNK expression was recorded after downregulating *hsc70-4* in *polyQ* background compared to the *polyQ* condition alone, whereas no significant fold change was seen when compared with the wild-type.

Cellular death ensues an increase in p-JNK, as it enhances the caspase activity (Borsello and Forloni, 2007; Perrin et al., 2009), so we looked for the real-time status of cellular death by using acridine orange (AO) live staining in eye discs. The eye disc of *GMR-GAL4:UAS-127Q* showed a drastic elevation in the AO puncta (Figure 6h) compared to *Oregon-R^+^* (Figure 6g), suggesting a significant increase in the number of apoptotic cells in the eye disc, which again confirms that elevated JNK expression leads to increased apoptosis. However, the level reduced significantly in *hsc70-4* downregulated condition almost to near normal (Figure 6i), which speculates a considerable rescue in the cellular apoptosis because of the back to the near-normal restoration of the p-JNK level after downregulation of *hsc70-4* in *polyQ* condition.

### Rescue in degenerated eye phenotype in Huntington model after downregulation of *hsc70-4*

Since the above studies were done using transgenic *Drosophila* having 127 glutamate residues to simulate the polyglutamine-associated disease condition like Huntington’s disease, so we validated it further by expressing mutant exon I of the *huntingtin* gene having 93 CAG repeats (*UAS-httex1PQ93*) in the eye discs of *Drosophila* in *GMR-*specified manner. As wild-type flies show normal eye pigmentation with a proper ommatidial arrangement (in Supplementary information 8A), these flies show age-wise progressive eye degeneration showing the loss of eye pigmentation along with the disrupted ommatidial arrangement (in Supplementary information 8B). The adults show almost complete loss of phototaxis response after the 10th day (Mallik and Lakhotia, 2009). Downregulating *hsc70-4* in *GMR-GAL4:UAS-httex1PQ93* background (in Supplementary information 8C) showed almost complete rescue in the loss of pigmentation and more compact arrangement of ommatidia. On the contrary, overexpressing the *hsc70-4* in *GMR-GAL4:UAS-httex1PQ93* background (in Supplementary information 8D) has an almost detrimental effect on the degenerated eye phenotype with higher pigmentation loss and more disrupted ommatidial arrangement.

These observations were further confirmed by the phototaxis assay performed on the 10-day-old flies (in Supplementary information 9). The population of *GMR-GAL4:UAS-httex1PQ93* flies showed more than a 50% reduction in the phototaxis response, and overexpressing *hsc70-4* did not show any significant changes. But approximately 70% of *GMR-GAL4:UAS-httex1PQ93* > *UAS-Hsc70-4RNAi* flies were found to show a positive phototaxis response, which validates the rescue in the eye phenotype. Thus, mutant *huntingtin* flies validate the rescue effect of the *hsc70-4* downregulation on the diseased eye phenotype.

## Discussion

Proteotoxic stress induced by neurodegenerative diseases is combated mainly by producing heat shock proteins, which act synergistically to either refold the misfolded proteins or degrade them. However, the response of all the heat shock proteins is not uniform and highly dependent on their type, disease, cell category, and brain regions. In the present study, we focused on the role of constitutively expressed heat shock cognate 70 in the progression of pathogenesis resulting from disrupted pathways. The data presented in this study show a differential response of heat shock cognate 70 isoforms to expanded PolyQ repeats, and *hsc70-4*, a *Drosophila* homolog for mammalian *HSPA8*, is maximally induced by the presence of PolyQ aggregates, and hence, its downregulation showed remarkable rescue.

Hsc70 is important in lysosomal-mediated autophagic degradation (Massey et al., 2006; Orenstein and Cuervo, 2010; Arias and Cuervo, 2011). However, its levels decrease in nigral neurons (Chu et al., 2009) and are enhanced in blood samples (Molochnikov et al., 2012) of Parkinson’s patients. Similar observations have been made for the stress-inducible Hsp70 (Calabrese et al., 2004; Njemini et al., 2007), as a reduction in the Hsp70 levels is observed in the olfactory neurons of Alzheimer’s patients (Getchell et al., 1995) which coincides with our observation about Hsp70 (data not shown). HSPs respond to cellular stress, but after some time, PolyQ aggregates hamper their ATPase activity by interacting with the ATPase domain (Carmichael et al., 2000; Krobitsch and Lindquist, 2000; Söti and Csermely, 2002; Soo et al., 2008). These findings emphasize that a standard paradigm cannot be applicable in all circumstances, and just an increase in a particular heat shock protein and its cognate forms may not be sufficient to clear the proteotoxicity because the expression of several other heat shock proteins and endogenous defense systems is also taken into account.

Several studies reported the neuroprotective role of HSPs, especially the *hsp70* gene family, in reducing the toxicity caused by PolyQ (Warrick et al., 1999; Chan et al., 2000; Evans et al., 2010; Voßfeldt et al., 2012; Kampinga and Bergink, 2016; Davis et al., 2020; Thiruvalluvan et al., 2020). Several other reports also showed the role of HSPA8 (Hsc70-4 in *Drosophila*) in the rescue from the proteinopathies in the case of neurodegenerative diseases (Bauer et al., 2010; Monsellier et al., 2015; Scior et al., 2018). However, our observation has shown that *hsc70-4* has been dysregulated in PolyQ-mediated neurodegeneration, and its downregulation rescues the disease pathogenicity. These observations are substantiated by reports suggesting that Hsc70-4 (HSPA8) is involved in aggravating the disease pathogenicity, as observed in tauopathies, where degradation of tau has been reduced by HSPA8 and decrement in this chaperonin level reduces the tau protein (Jinwal et al., 2009, 2013; Fontaine et al., 2015). In another report, Hsc70-4 interacts with the ataxin-3 protein in SCA-3, which aggravates the disease phenotype (Johnson et al., 2020). The proteostasis networks collapse as these aggregates start disrupting the ubiquitin–proteasome systems, further increasing the aggregate burden (Balch et al., 2008; Labbadia and Morimoto, 2013; Ortega and Lucas, 2014). A recent study on CLN4-mediated neurodegeneration showed that reducing the gene dosage of *hsc70-4* leads to the attenuation of the CLN4 diseased phenotype by restoring normal membrane trafficking (Imler et al., 2019).

The protection conferred by the immune response can sometimes be overruled by their erroneous activation, which aggravates the disease phenotype (Blach-Olszewska and Leszek, 2007). The humoral immunity branch of the innate immune response in *Drosophila* is mediated by the activation of the Toll and IMD pathway by the homologs of the NF-κB transcription factor. Studies have shown that hyperactivated immune response is one of the critical hallmarks of *polyQ*-mediated neurodegeneration (Ellwardt and Zipp, 2014; McManus and Heneka, 2017; Gelders et al., 2018). Hsp70 regulates NF-κB activation by inhibiting IκB-α phosphorylation (Huang et al., 2013; Chang et al., 2014; Wang et al., 2017; Yi et al., 2018). However, Hsc70 is involved in the activation of NF-κB *via* promoting phosphorylation of IκB-α and maintaining the NF-κB/IκB-α/IKKβ complex, thus playing a crucial role in the cleavage of inhibitor domain at the time of NF-κB activation (Sheppard et al., 2014; Kumada et al., 2019). The decrease in Hsc70-4 in *GMR-GAL4:UAS-127Q* progenies and consequent reduction in the expression of Relish confirm that Hsc70 regulates the activation of Relish. This inference was further substantiated by the physical interaction of Hsc70 and Relish. PolyQ aggregates interact with Relish to co-translocate it into the nucleus (Takano and Gusella, 2002; Khoshnan et al., 2004). The interaction of Hsc70 with aggregate protein in neurodegenerative diseases has revealed the nature and the probable purpose of the interaction in different neurodegenerative conditions (Monsellier et al., 2015; Kim et al., 2016; Imler et al., 2019; Johnson et al., 2020). Therefore, the possible involvement of Hsc70-4 in the PolyQ-mediated immune upregulation was speculated, and confirmed as the transcripts of *relish*, and several other AMPs, such as *attacin, cecropin, drosocin, drosomycin*, and *diptericin*, were reduced after downregulation of *hsc70-4* in *polyQ* background. Reports mention the interaction of Hsc70 with Relish in various inflammatory conditions (Sheppard et al., 2014; Wang et al., 2017), but regarding neurodegenerative diseases, especially in the case of *polyQ*-mediated neurodegeneration, this interaction has not been shown. The possible mechanism for the regulation is the unwanted crosstalk between Hsc70-4 and Relish during their interaction with the PolyQ aggregates, leading to Relish’s overactivation.

PolyQ aggregates accumulate in the axons, leading to the titration of several motor and cargo proteins, which hamper the normal growth and proper organelles distribution in the axonal processes. Thus, it affects the various molecular transport across the axons (Gunawardena et al., 2003), leading to abnormal axonal connections as observed in the *polyQ* condition. The correct ommatidial arrangement is highly dependent on cadherins maintained by β-catenin. These cross-linking molecules get disrupted in *polyQ* conditions, thus disrupting the ommatidial arrangement (Godin et al., 2010; Yadav and Tapadia, 2013). Additionally, the loss of proper axonal growth and development, along with disrupted ommatidial arrangement, leads to structural and functional loss of the eye, which gets rescued upon downregulating *hsc70-4*. Previous studies show that the negative regulation of β-catenin by NF-κB (Ma and Hottiger, 2016) might be causing its dysregulation in *polyQ-*expressed conditions. Since downregulation of *hsc70-4* normalizes the hyperactivated Relish (NF-κB), reduced Relish expression might contribute toward the structural rescue in the *polyQ*-expressing eyes.

The p-JNK, an activated form of JNK protein activated by MAP Kinases, leads to ameliorating the inflammatory response and gets overexpressed in any cellular insult. It is also overexpressed in the case of PolyQ toxicity leading to a significant increase in cellular apoptosis induced by the hyperactivated immune response (Liu and Lin, 2005; Jing and Anning, 2005; Dubey and Tapadia, 2018). Studies have shown the antiapoptotic effect of Bcl-2-associated athanogene3 (BAG3) in myocardium functions by associating with Hsc70 and JNK (Zhao et al., 2021). So, downregulating *hsc70-4* in the *GMR-GAL4:UAS-127Q* background reduces the level of activated JNK, which decreases the level of immune-induced cellular apoptosis.

We hypothesize that PolyQ aggregates may increase the levels of Hsc70-4, which may increase Relish activation and indirectly or directly increase the JNK activity. This mechanism could pave the path for newer therapeutic interventions to prevent human neurodegenerative diseases. Also, this study, among several others, helps to illuminate the unconventional role of exacerbating the *polyQ*-mediated neurodegeneration by dysregulated *hsc70-4*. Finally, this provides evidence to link heat shock cognate 70, neurodegeneration, and innate immune response in developing disease conditions.

## Data availability statement

The original contributions presented in the study are included in the article/Supplementary material, further inquiries can be directed to the corresponding author.

## Author contributions

SR performed the experiments. Both authors conceived and designed experiments, analyzed the data, and wrote and reviewed the manuscript.
