# Supplementary material for: Hsc70-4 aggravates PolyQ-mediated neurodegeneration by modulating NF-κB mediated immune response in Drosophila
Source: Front Mol Neurosci. 2022 Nov 8;15:857257. doi: 10.3389/fnmol.2022.857257 (PMC9678916; doi:10.3389/fnmol.2022.857257)

**Supplementary Information:**

**S1. Table 1:** List of Forward and Reverse Primer Sequences

| | |
|---|---|
| *Rp49* F | TTGAGAACGCAGGCGACCGT |
| *Rp49* R | CGTCTCCTCCAAGAAGCGCAAG |
| *Rel* F | GAAAAACCCGTGAGTCATCTC |
| *Rel* R | AACGCCGAAACTAACGCCAG |
| *Att-A* F | GATGGACGTGCTAATCTCTG |
| *Att-A* R | GGCTTAGCCGAAATGATGAG |
| *Dipt-A* F | AGTTCACCATTACCGTCGCC |
| *Dipt-A* R | GTAGGTGTAGGTGCTTCCCA |
| *Drm*F | TTGTCCTGTCCGCTGTCTTG |
| *Drm* R | GCATTTTAGACTGGGGCTGC |
| *Dro* F | TTTTCCTGCTGCTTGCTTGC |
| *Dro* R | GGCAGCTTGAGTCAGGTGAT |
| *Def* F | CGTGGCTATCGCTTTTGCTC |
| *Def* R | GAGTAGGTCGCATGTGGCTC |
| *CecC*F | CGGGATGCAACCATTCAAGG |
| *CecC*R | CCACATTGGCGGCCTGTT |
| *Hsc70.1* F | GAGATAACAACAGCCTGGGTAAG |
| *Hsc70.1* R | CTCAAATGGTTGGACCCGAG |
| *Hsc70.2* F | TCCGAAACCGAACGCTCCAAGG |
| *Hsc70.2* R | TGGCACTCTTTCAGGTGACTC |
| *Hsc70.3* F | ACCAGAACCGCCTGACGCCC |
| *Hsc70.3* R | GGGATCGGCATCGGGATTCTGT |

| | |
|---|---|
| *Hsc70.4* F | TGCCCTGGAGCGTTCGACCAA |
| *Hsc70.4* R | GCATACCTCCGGGACCGCC |
| *Hsc70.5* F | ATAAGCTTCTCGGCTCCTTTACA |
| *Hsc70.5* R | TATCGTGGACTATGCTCTCGCCC |
| *Hsf*F | TGGCCAGCTTCATAAGGCAA |
| *Hsf*R | ACCCCGCATGACTTTCACAT |

**S2.** Heat map plot for the means of fold change (± SEM) in the transcript levels of hsc*70-1, hsc70-2*, *hsc70-3, hsc70-4,* and *hsc70-5*. Quantitative RT-PCR analysis of the expression was normalized to *rp49*.

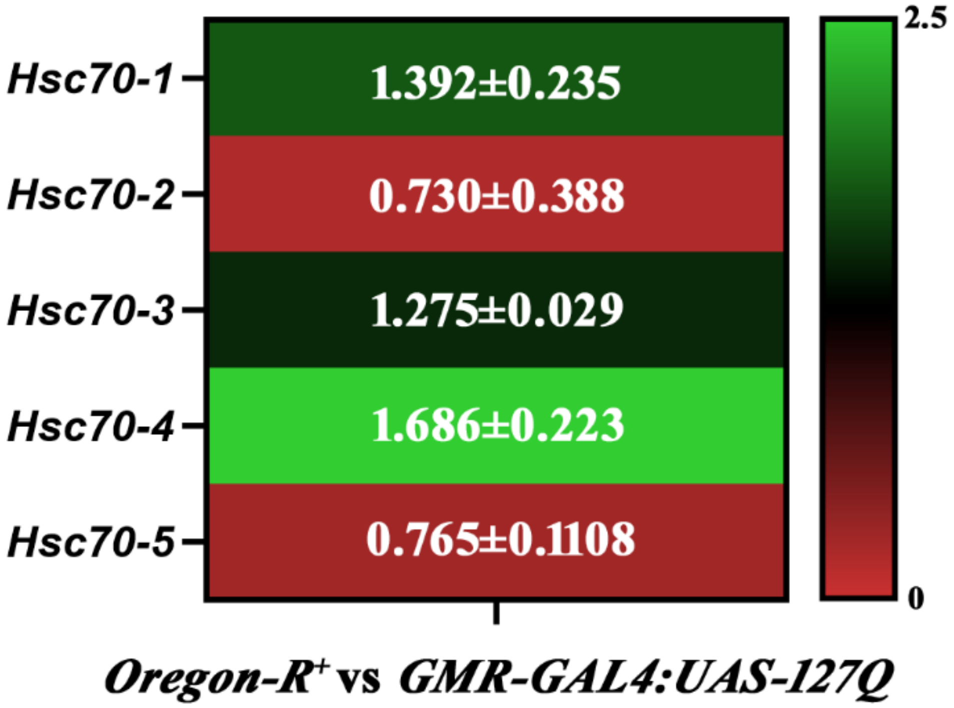

**S3.** Graph showing the fold change in the *hsc70-4* transcript level in the respective genotypes. Both RNAi lines showed a significant reduction in the expression of the transcript upon knocking down the *hsc70-4* in *polyQ*-expressed conditions. Quantitative RT-PCR analysis of the expression of *hsc70-4* was normalized to *rp49*.

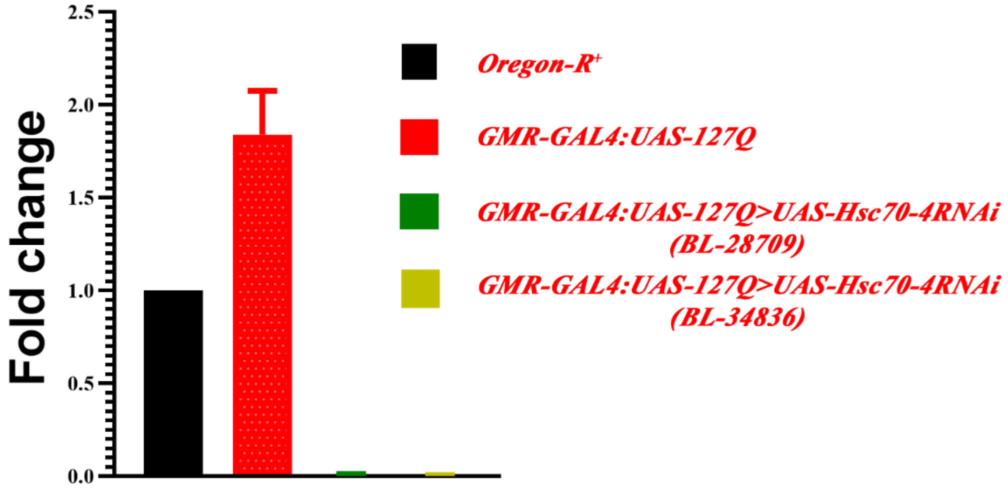


**S4.** Full-length Western Blots along with the protein ladder, the cropped band images of which are shown in Figure1 B. Blot A shows the changes in the protein expression of the respective genotypes (upper band in blot-A shows the ~71kDa Hsc70 protein while the lower band shows the inducible Hsp70 expression) and blot B shows the expression of Beta-tubulin in the same (used as an internal control for data normalization). In order to show the change in the expression of Hsc70 protein, only the upper bands were used to measure the relative band intensity and shown as the fold changes in Hsc70 protein in Figure1, C of the result section.

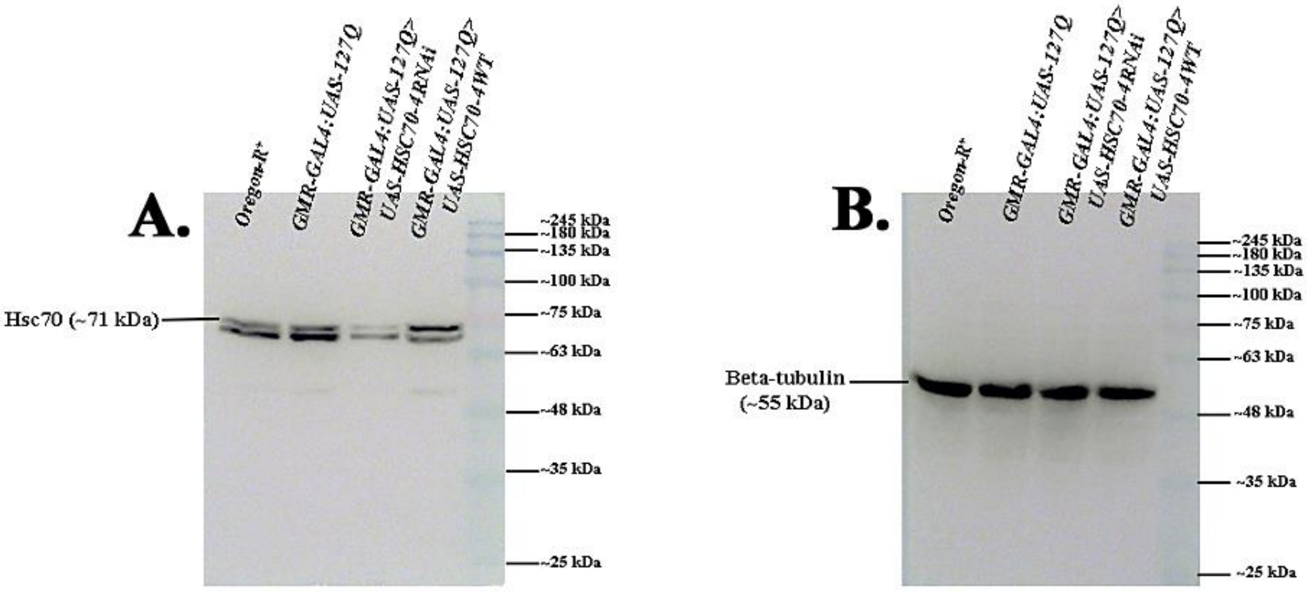

Histogram showing the fold changes in the inducible-Hsp70 (~68kDa) in Figure C. Validation was done by calculating the fold changes in the Hsp70 from three independent experiments, and statistical significance was determined by using One-way ANOVA followed by Tukey's multiple comparisons test (* indicates the significance at a p-value of ≤0.05 and ** indicates the significance at a p-value of ≤0.001).

C.

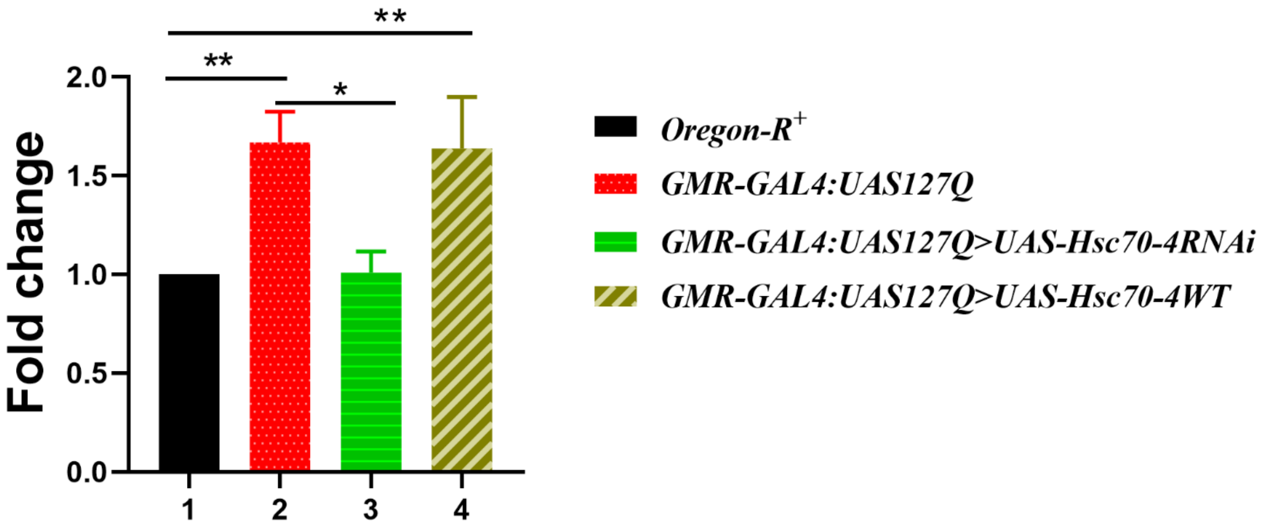


**S5.** Disc large1(Dlg) is required to maintain the membrane's apicobasal polarity and has a suspected role in cell-cell adhesion. Dlg is strictly located on the membrane of rhabdomeres in *Oregon-R*$^+$, thus marking a precise shape of ommatidia in the eye disc (in Fig S5, A1). The usual rhabdomere arrangement and ommatidia number are also reduced in the case of *GMR-GAL4:UAS-127Q*, as the membrane boundaries are shrunk and fused (marked by arrows), leading to the reduced number of rhabdomere and hence, the disrupted ommatidia structure (in Fig S5, A2).

After downregulating *hsc70-4*, the rhabdomere boundary disruption is restored, resulting in the rescued ommatidia structure (in Fig S5, A3). In contrast, upon overexpressing *hsc70-4*, a thick and diffused boundary appears, which dictates a more severe disruption of the ommatidia compared to the *GMR-GAL4:UAS-127Q* (in Fig S5, A4). After this, the number of rhabdomeres within ommatidia was counted, and was found that an average of seven rhabdomeres are present in the wild-type. It was reduced up to an average of four rhabdomeres per ommatidium in the *GMR-GAL4:UAS-127Q* and was reduced to an average of three rhabdomeres per ommatidium after over-expressing *hsc70-4* in the same condition. However, downregulation *hsc70-4* rescued

this disruption of ommatidia, via rescuing the rhabdomeres to a mean of five or more in each ommatidium in the *polyQ*-driven background.

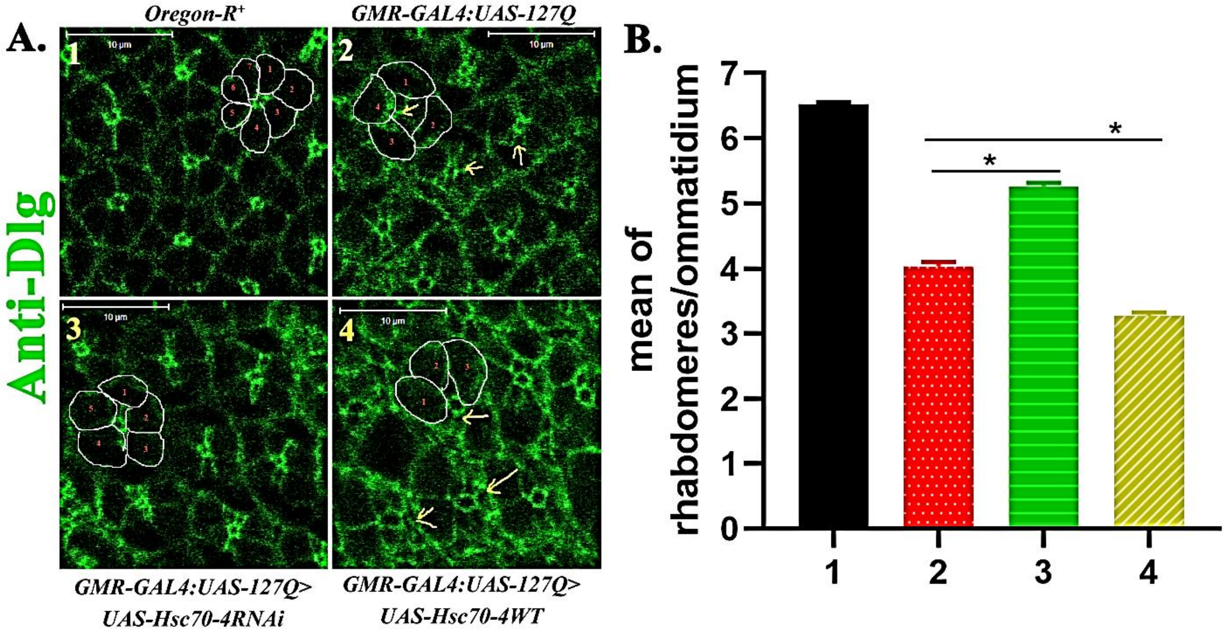


**Figure S5. (A)** The panel shows the staining pattern of Dlg1 (Disc large1), marking the membrane in the ommatidia of the respective genotype. Each image in the panel shows the section image of the eye disc of the 3$^{rd}$ instar larvae of **1.** *Oregon-R*$^{+}$, **2.** *GMR-GAL4:UAS-127Q*, **3.** *GMR-GAL4:UAS-127Q>UAS-Hsc70-7RNAi*, **4.** *GMR-GAL4:UAS-127Q>UAS-Hsc70-4WT*. In this panel, white boundaries mark the rhabdomeres in an ommatidial unit of each image and their numbers are labeled (in red) within it. In images 2 and 4, arrows (in yellow) demarcate the diffused boundary of the rhabdomere, which confers the ommatidia disruptions. (Scale bar 10μm). **(B)** Histogram showing the mean number of rhabdomeres per ommatidium in the genotypes shown in the panel. Each data point was based on an examination of ≥50 ommatidia from n≥10 eye discs. Error bars represent the SEMs.The statistical significance was determined by One-way ANOVA, followed by Sidak multiple comparison analysis (* indicates the significance at a p-value of ≤0.001).

**S6.** The figure shows the surface rendering of the 3D projection from the Z-stack of the eye disc of *GMR-GAL4:UAS-127Q* 3rd instars. The Panel shows the cropped section of projection focusing on the PolyQ aggregate (in grey), Relish (in green), and Hsc70 (in red). The Panel portrays how the PolyQ aggregates sequester Hsc70 and relish. Here, images a and e show sliced PolyQ aggregates, which help visualize the signal inside the aggregate's boundary. The sequestration of Relish and Hsc70 is visualized separately in c; g and d; h, respectively, and the co-sequestration of Relish and Hsc70 is shown in b and f. (Scale Bar 200 pixel).

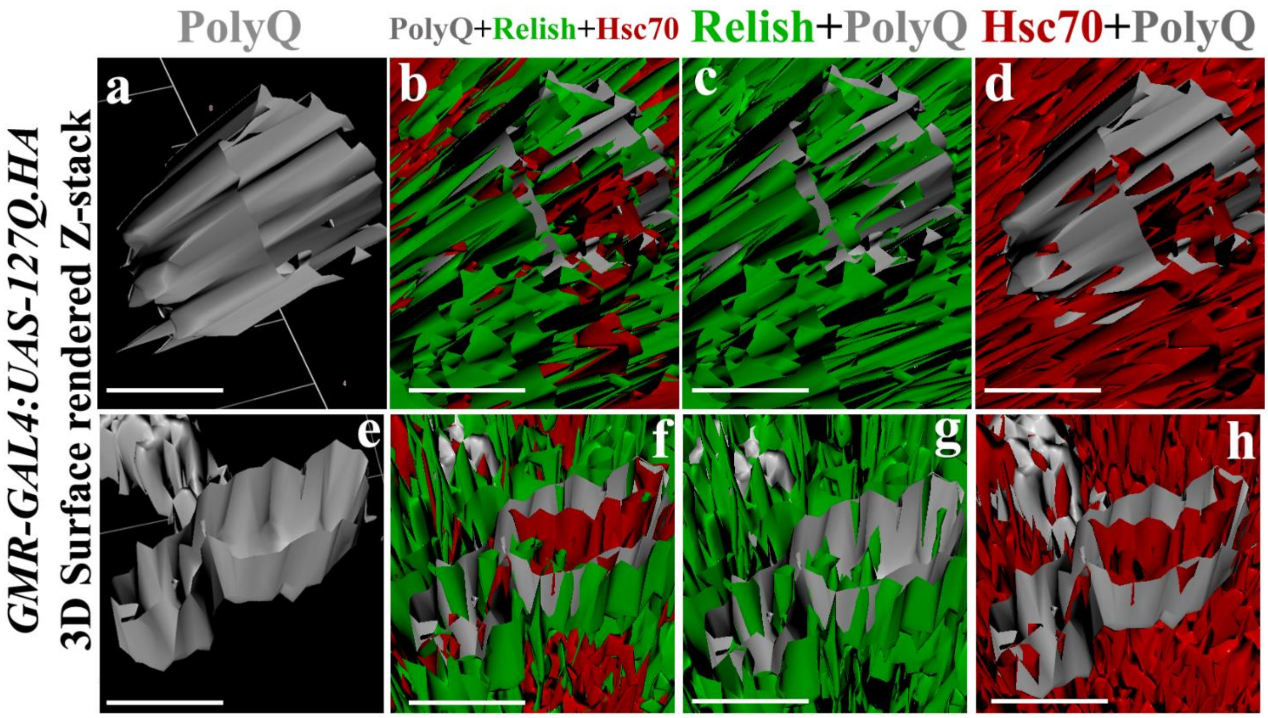

**S7.** A graph showing the change in the expression level of p-JNK in Figure 5, a-c. Quantitative analysis was done by observing three independent experiments' mean intensity/pixel of the immune-fluorescence images showing p-JNK expressions in the different genotypes. The statistical significance was determined by One-way ANOVA, followed by Sidak multiple comparison analysis (ns indicates insignificant change at a p-value ≤0.05 and * indicates the significance at a p-value of ≤0.05).

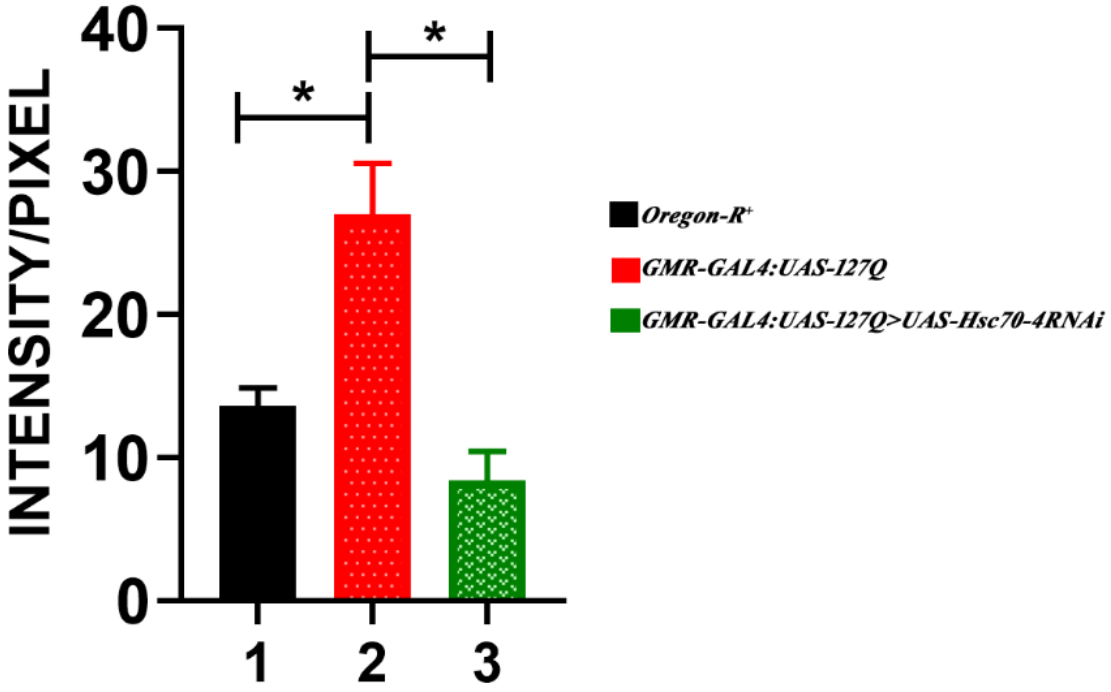

**S8. Degenerated eye phenotype was rescued after downregulation of *hsc70-4*.** (a) Eye morphology of *Oregon-R*$^{+}$, (b) *GMR-GAL:UAS-httex1PQ93* expresses 93CAG repeats in exon 1, which results in the loss of eye pigmentation and disruption of ommatidia, (c) *GMR-GAL:UAS-httex1PQ93>UAS-Hsc70-4RNAi* restores the loss of pigmentation and the ommatidial arrangement in comparison to *GMR-GAL:UAS-httex1PQ93*, (d) *GMR-GAL:UAS-httex1PQ93>UAS-Hsc70-4WT* showed a severe loss of pigmentation and disruption in ommatidial arrangement, (Scale bar 100µm).

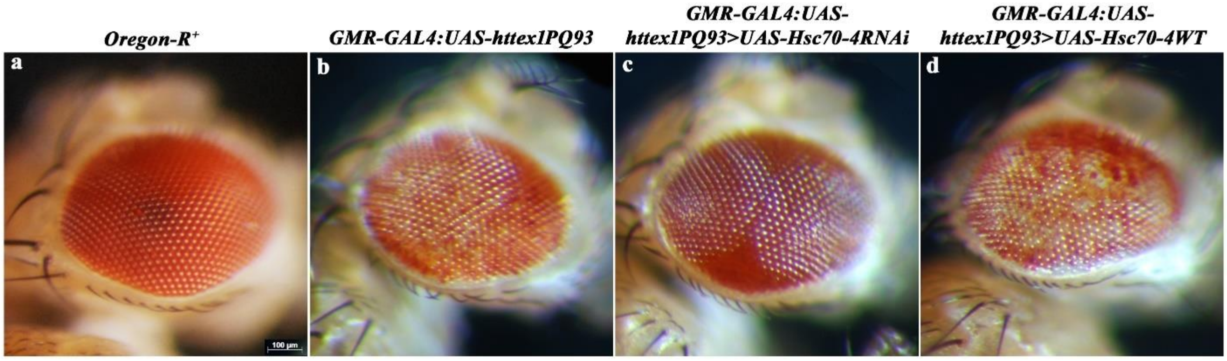


**S9.** The Graph shows the phototaxis response in 10-day-old flies of *Oregon-R*$^{+}$, *GMR-GAL4:UAS-httex1PQ93*, *GMR-GAL4:UAS-httex1PQ93>UAS-Hsc70-4RNAi* and *GMR-GAL4:UAS-httex1PQ93>UAS-Hsc70-4WT*. Three independent experiments were performed with n ≥ 100, and statistical significance was determined by using one-way ANOVA followed by Sidak multiple comparison analysis (ns indicates insignificant change at a p-value ≤0.05 and * indicates significance at a p-value of ≤0.05).

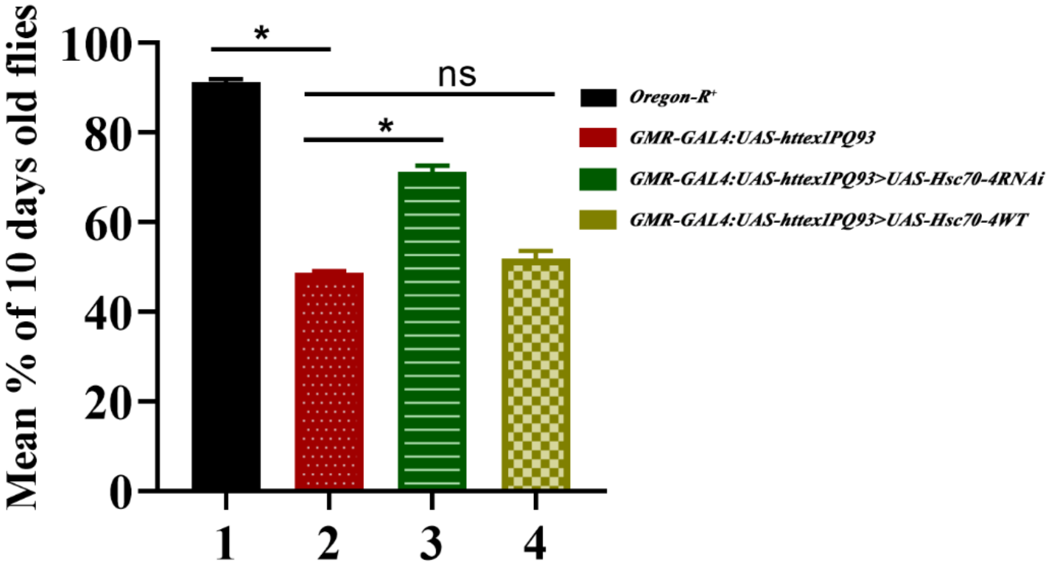

Supplement: Supplementary file 1 [file Data_Sheet_1.PDF]
